# Transition from an M1 to a mixed neuroinflammatory phenotype increases amyloid deposition in APP/PS1 transgenic mice

**DOI:** 10.1186/1742-2094-11-127

**Published:** 2014-07-25

**Authors:** Erica M Weekman, Tiffany L Sudduth, Erin L Abner, Gabriel J Popa, Michael D Mendenhall, Holly M Brothers, Kaitlyn Braun, Abigail Greenstein, Donna M Wilcock

**Affiliations:** 1 Sanders-Brown Center on Aging, University of Kentucky, Lexington, KY 40536, USA; 2 Department of Physiology, University of Kentucky, Lexington, KY 40536, USA; 3 Department of Epidemiology, University of Kentucky, Lexington, KY 40536, USA; 4 Department of Molecular and Cellular Biochemistry, University of Kentucky, Lexington, KY 40536, USA

**Keywords:** Inflammation, Alzheimer’s disease, Microglia, Beta-amyloid

## Abstract

**Background:**

The polarization to different neuroinflammatory phenotypes has been described in early Alzheimer’s disease, yet the impact of these phenotypes on amyloid-beta (Aβ) pathology remains unknown. Short-term studies show that induction of an M1 neuroinflammatory phenotype reduces Aβ, but long-term studies have not been performed that track the neuroinflammatory phenotype.

**Methods:**

Wild-type and APP/PS1 transgenic mice aged 3 to 4 months received a bilateral intracranial injection of adeno-associated viral (AAV) vectors expressing IFNγ or green fluorescent protein in the frontal cortex and hippocampus. Mice were sacrificed 4 or 6 months post-injection. ELISA measurements were used for IFNγ protein levels and biochemical levels of Aβ. The neuroinflammatory phenotype was determined through quantitative PCR. Microglia, astrocytes, and Aβ levels were assessed with immunohistochemistry.

**Results:**

AAV expressing IFNγ induced an M1 neuroinflammatory phenotype at 4 months and a mixed phenotype along with an increase in Aβ at 6 months. Microglial staining was increased at 6 months and astrocyte staining was decreased at 4 and 6 months in mice receiving AAV expressing IFNγ.

**Conclusions:**

Expression of IFNγ through AAV successfully induced an M1 phenotype at 4 months that transitioned to a mixed phenotype by 6 months. This transition also appeared with an increase in amyloid burden suggesting that a mixed phenotype, or enhanced expression of M2a and M2c markers, could contribute to increasing amyloid burden and disease progression.

## Background

First described by Alois Alzheimer in 1907, Alzheimer’s disease (AD) is a progressive, neurodegenerative disease characterized pathologically by the presence of amyloid plaques formed by amyloid-beta (Aβ) peptide aggregates and neurofibrillary tangles composed of hyperphosphorylated and aggregated tau protein [1]. Alois Alzheimer also described inflammation in the form of microglia surrounding amyloid plaques. Numerous studies have shown that microglial activation results from a reaction to Aβ in AD [2-5]. The overall role of neuroinflammation in AD remains relatively unknown.

Peripheral macrophages have been extensively characterized and shown to have distinct phenotypes dependent on their stimuli. The M1 phenotype, or classical activation, is associated with defense and attack and induces the release of pro-inflammatory cytokines such as IL-1β, TNFα, IL-12 and IL-6 [6,7]. The M2 phenotypes are termed alternative activation and include the M2a, M2b and M2c states. The M2a phenotype is characterized by wound healing and high levels of arginase 1 (ARG1) and the chitinase-like protein YM1 [8]. The M2b phenotype is a combination of an M1 and M2a state and is associated with high levels of CD86 and the Fc gamma receptors 1 and 3 (FcγR1 and FcγR3) [8]. Finally, the M2c phenotype is a deactivation state accompanied by high levels of transforming growth factor beta and sphingosine kinase 1 [6,9]. It has more recently been shown that microglia are also capable of expressing most, if not all, of these inflammatory mediators under the correct conditions.

The interferon family of cytokines has been shown to be increased in human AD tissue and in the APP/PS1 mouse model, and IFNγ is the main stimulant for microglia to produce an M1 phenotype by binding its receptor, increasing STAT1α, and increasing transcription of several M1 cytokine genes [10,11]. Some studies have used IFNγ to induce an M1 phenotype, with one study showing that classical activation of microglia produces a decrease and another reporting an increase in amyloid burden [12,13]. Other studies have shown that inducing an M1 neuroinflammatory phenotype by introducing TNFα, IL-1β, or lipopolysaccharide (LPS) into the brain lowers amyloid burden [14-17]. However, these studies do not measure or track the different neuroinflammatory phenotypes along with the changes in amyloid burden over long periods of time. Additionally, engaging the immune system in the brain using anti-Aβ immunotherapy has been shown to be extremely efficacious in lowering amyloid load [18,19].

In order to better understand the long-term effects of an M1 neuroinflammatory phenotype we bilaterally injected an adeno-associated viral (AAV) vector expressing IFNγ into the frontal cortex and hippocampus of wild-type and APP/PS1 mice to induce an M1 phenotype and determine the effects on Aβ levels at 4 and 6 months after injection. We found that IFNγ induced an M1 phenotype at 4 months but transitioned to include an M2 phenotype by 6 months. The transition from an M1 to a mixed phenotype did not ameliorate Aβ levels at 6 months. Instead, Aβ levels were significantly higher, suggesting that a mixed phenotype could accelerate the disease process.

## Methods

### Animals

Female and male wild-type (WT) and APP/PS1 transgenic mice (C57BL6 mice carrying human APPSwe and PS1-dE9 mutations) [20] were bred in house and aged for 3 to 4 months. Mice were randomly placed into one of eight groups based on AAV serotype 8 (AAV-8) injection, genotype and survival. Fifteen WT mice were assigned to receive either AAV-8 expressing green fluorescent protein (GFP) (GFP-AAV) for 4 months (n = 2 females, 1 male) or 6 months (n = 3 females, 3 males), or AAV-8 expressing IFNγ (IFNγ-AAV) for 4 months (n = 2 females, 1 male) or 6 months (n = 2 females, 1 male). Twenty APP/PS1 mice were assigned to receive either GFP-AAV for 4 months (n = 2 females, 2 males) or 6 months (n = 4 females, 4 males) or IFNγ-AAV for 4 months (n = 2 females, 1 male) or 6 months (n = 2 females, 3 males). This study was approved by the University of Kentucky Institutional Animal Care and Use Committee and conformed to the National Institutes of Health Guide for the Care and Use of Animals in Research.

### Adeno-associated virus preparation

The vector for preparing recombinant AAV was constructed by ligating the 1349 bp *Eco*RI/*Sal*I fragment carrying the IRES-GFP from pSMPUW-IRES-GFP (Cell Biolabs, San Diego, CA, USA) into *Eco*RI/*Sal*I-digested pZac2.1 (gift of Dr Paul Murphy, University of Kentucky) to create ViCo1.28. The IFNγ insert was prepared by PCR amplification of cDNA clones obtained from Open Biosystems (GE Healthcare, Dharmacon RNAi and gene expression, Piscataway, NJ, USA). The IFNγ PCR primer sequence used was CCCGCTAGCTCTGAGACAATGACCACCGCGGACCCCGAATCAGCAGCGA. The Open Biosystems Catalogue number for the sequence is MMM1013-99829104. The clone ID is 8733812 and the accession is BC119063. The primers introduced an *Nhe*I site at the 5’-end and a *Sac*II site at the 3’-end of the IFNγ gene to facilitate cloning into the corresponding sites in ViCo1.28. The fidelity of each clone was confirmed by DNA sequence analysis.

AAV8 coat protein-pseudotyped AAV2 viruses were prepared by co-transfecting 10 T225 culture flasks of 293LTV cells (Cell Biolabs) with 250 μg pAAV2/8 (obtained from the University of Pennsylvania Viral Core), 500 μg pAdΔF6 (gift of Dr Paul Murphy) and, individually, 250 μg of each cytokine clone using 5 mg polyethyleneimine to enhance DNA uptake. After 3 days, the cells were harvested, washed, suspended in 13 ml 150 mM NaCl, 50 mM Tris · Cl pH 8.4, 0.5% deoxycholate and 50 U/ml of benzonase and incubated at 37°C for 30 minutes. An additional 2.8 ml 5 M NaCl was added and the incubation was continued for another 30 minutes at 45°C. The cell suspension was then subjected to four freeze/thaw cycles (30 minutes at −80°C/30 minutes at 45°C). The lysate was then partially clarified by centrifugation at 18,500 × g for 10 minutes at 20°C. The supernatant was laid on top of an iodixanol step gradient and centrifuged at 350,000 × g for 1 hour at 18°C. The interface between the 40% and 54% iodixanol layers was withdrawn and spin-purified and concentrated using four washes with PBS in an Amicon Ultra-15 100,000 MWCO spin concentrator (Thermo Fisher, Rockford, IL)). The virus preparation was then titered using real-time PCR with primers directed against the cytomegalovirus (CMV) promoter region of the DNA encapsulated in the virions.

### Bilateral intracranial injection

On the day of surgery, mice were anesthetized with isoflurane and placed into a stereotaxic apparatus (51733D digital dual manipulator mouse stereotaxic frame; Stoelting Co., Wood Dale, IL, USA). A midsaggital incision was used to expose the skull. Using a dental drill mounted on the stereotaxic frame, a total of four burr holes were made, one hole for each frontal cortex and hippocampus. The following coordinates were used from bregma: frontal cortex, anteroposterior, +2.0 mm, lateral ± 2.0 mm; hippocampus, anteroposterior −2.7 mm, lateral ± 2.5 mm. These have been previously established by the laboratory using dye injections to confirm appropriate placement. A 26 gauge needle attached to a 10 μL Hamilton syringe (Hamilton, Reno, NV, USA) containing the AAV at a concentration of 2 × 10^9^ genomes/μL to be injected was lowered 3.0 mm ventral to bregma, and a 2 μL injection was made over a 4-minute period. The incision was cleaned and sutured. Sutures were removed 2 weeks after surgery. The virus was injected at the maximum titer possible based on the concentration supplied so as to achieve the greatest expression of IFNγ.

### Tissue processing

After injection with a lethal dose of Beuthanasia-D, mice were perfused intracardially with 25 mL normal saline. Brains were removed rapidly and bisected in the midsaggital plane. The left side of the brain was immersion fixed in 4% paraformaldehyde. The right side was dissected with the frontal cortex and hippocampus being isolated and flash frozen in liquid nitrogen and then stored at −80°C. The left hemibrain was passed through a series of 10, 20 and 30% sucrose solutions for cryprotection. Using a sliding microtome, 25 μm frozen horizontal sections were collected and stored free floating in 1XDPBS containing sodium azide at 4°C.

### Immunohistochemistry

Six floating sections spaced 300 μm apart spanning the injection site (1800 to 3600 μm ventral to bregma) were immunostained by using commercially available antibodies against Aβ (Rabbit polyclonal Aβ1-16, Life Technologies, Carlsbad, CA, USA), CD11b (Rat monoclonal, AbD Serotec, Raleigh, NC, USA), and glial-fibrillary acidic protein (GFAP; Rabbit polyclonal, Dako, Denmark). Immunohistochemistry was performed as previously described [21]. Briefly, sections were quenched for endogenous peroxidase, blocked and permeabalized. They were then incubated overnight in primary antibody at 4°C (Aβ 1:3000, CD11b 1:3000, GFAP 1:10000). After washing, sections were incubated for 2 hours in the appropriate biotinylated secondary antibody (goat anti-rabbit IgG for Aβ and GFAP, goat anti-rat for CD11b, all 1:3000; Vector Laboratories, Burlingame, CA, USA). Sections were then washed and incubated for 1 hour in ABC. For CD11b and GFAP, color development was performed using the DAB substrate kit with Nickel (Vector Laboratories). For Aβ, color development was performed using powder DAB (Sigma, St Louis, MO, USA).

Stained sections were mounted, air dried overnight, dehydrated and coverslipped in DPX (Electron Microscopy Sciences, Hatfield, PA, USA). Immunohistochemical analysis was performed by measuring percent area occupied by positive stain using the Nikon Elements BR image analysis system (Melville, NY, USA) as described previously [22].

### Quantitative real-time PCR

The Trizol plus RNA purification system (Life Technologies) was used to extract RNA from the frozen right hippocampus according to the manufacturer’s instructions. RNA was quantified using the Biospec nano spectrophotometer (Shimaduz, Japan). cDNA was produced using the cDNA High Capacity kit (Life Technologies) according to the manufacturer’s instructions. Real-time PCR was performed using the Fast TaqMan Gene Expression assay (Life Technologies). In each well of a 96-well plate, 0.5 μL cDNA (100 ng, based on the RNA concentrations) was diluted with 6.5 μL RNase-free water. One microliter of the appropriate gene probe was added along with 10 μL of the Fast TaqMan to each well. Target amplification was performed using ViiA7 (Applied Biosystems, Grand Island, NY, USA). The thermal cycling conditions include a holding stage at 95°C followed by 40 cycles of denaturation at 95°C for 1 second and annealing/primer extension at 60°C for 20 seconds. All genes were normalized to 18S rRNA. We determined the fold change for mice receiving IFNγ-AAV compared with mice receiving GFP-AAV using the ^–ΔΔCt^ method [23]. Table 1 shows the genes tested along with their PMID and Taqman ID.

**Table 1:** Genes for real-time PCR

**Gene of interest**	**PMID**	**Taqman ID**
IL-6	NM_031168.1	Mm00446190_m1
IL-1β	NM_008361.3	Mm00434228_m1
IL-12a	NM_008351.2	Mm00434165_m1
IL-12b	NM_008352.2	Mm00434174_m1
ARG1	NM_007482.3	Mm00475988_m1
YM1 (Chil3)	NM_009892.2	Mm00657889_mH
CD86	NM_019388.3	Mm00444543_m1
FcγR1	NM_010186.5	Mm00438874_m1
FcγR3	NM_010188.5	Mm00438882_m1
TGFβ1	NM_011577.1	Mm01178820_m1
SPHK1	NM_011451.3	Mm01252544_m1

ARG, arginase; FcγR, Fc gamma receptor; IL, interleukin; SPHK, sphingosine kinase; TGF, transforming growth factor.

### ELISA measurement

Protein for Aβ and IFNγ analysis was extracted from the right frontal cortex in 1XDPBS with complete protease and phosphatase inhibitor (Pierce Biotechnology Inc., Rockford, IL, USA). The samples were centrifuged at 10,000 × g at 4°C for 15 minutes. The supernatant was removed and labeled the “soluble” extract. The pellet was homogenized in 250 μL 70% formic acid and centrifuged at 10,000 × g at 4°C for 1 hour. The supernatant was removed and neutralized 1:20 with 1 M Tris–HCl and brought to a pH of 7 with HCl. This was labeled the “insoluble” extract. Protein concentrations for the soluble and insoluble extracts were determined using the BCA protein assay kit (Pierce Biotechnology Inc.) according to the manufacturer’s instructions. The Meso-Scale Discovery multiplex ELISA system was used to measure Aβ1-38, Aβ1-40, and Aβ1-42 levels in the soluble and insoluble extracts (MSD, Gaithersburg, MD, USA). The Meso-Scale Discovery Mouse Proinflammatory 7-Plex kit (MSD) was used to measure IFNγ levels in the soluble extract. All steps were followed according to the manufacturer’s directions except for the sample incubation time which was left overnight.

### Analysis

Data are presented as mean ± SEM. We used two-way analysis of variance (ANOVA) based on the factors treatment (GFP-AAV or IFNγ-AAV injection) and survival time (4 or 6 months) to evaluate main effects and treatment-by-time interaction within genotype (WT and APP/PS1). No statistical comparisons were made across genotype. Because treatment effects at each time point were of interest *a priori*, data are presented by treatment and time point whether or not the treatment-by-time interaction is significant.

Due to a high proportion of zero values, we used nonparametric two-way ANOVA on ranks to evaluate distributional differences in ELISA-determined IFNγ protein levels. Similarly, we used two-way ANOVA on ranks to evaluate differences in gene expression (measured by fold change from GFP) as these data did not meet parametric ANOVA assumptions. Ranks were assigned using the RANK procedure, and ANOVA was performed using the GLM procedure in SAS/STAT 9.3® (SAS, Cary, NC, USA). Statistical significance was set at *P* < 0.05.

## Results

**Figure 1:**
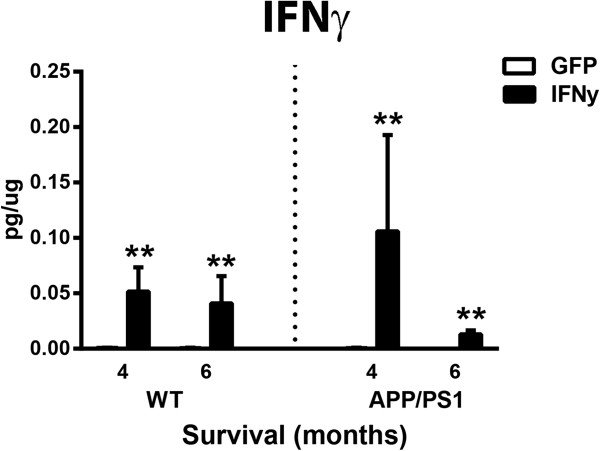
**Protein analysis confirms IFNγ overexpression by adeno-associated virus.** **P < 0.01 for IFNγ-AAV compared to GFP-AAV at that time point and genotype. AAV, adeno-associated virus; IFN, interferon; GFP, green fluorescent protein; WT, wild-type.

To determine that the IFNγ-AAV resulted in expression of IFNγ we performed an ELISA measurement. Measurement of IFNγ in the protein extracted from the right frontal cortex showed a significant treatment effect such that mice injected with IFNγ-AAV had a 5,000-fold increase in IFNγ levels at 4 and 6 months in both WT and APP/PS1 mice compared to mice receiving GFP-AAV (Figure 1).

**Figure 2:**
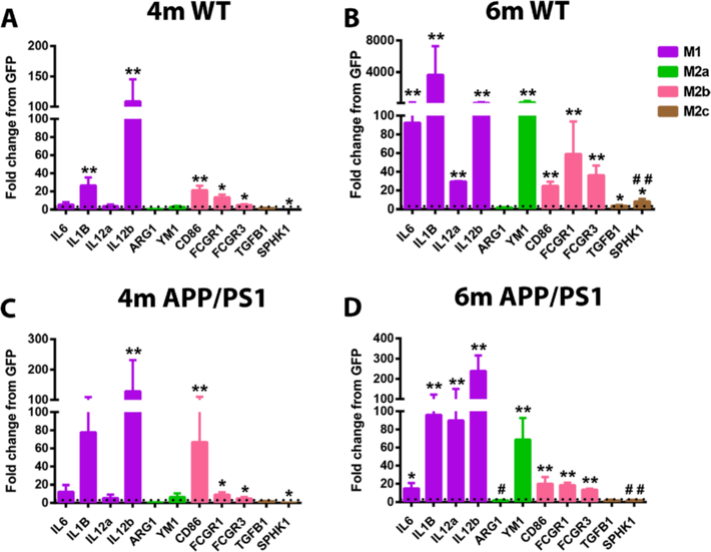
**IFNγ induces an M1 neuroinflammatory phenotype.** Relative gene expression for genes representative of the M1, M2a, M2b, and M2c phenotypes. Data are shown as fold-change relative to mice receiving GFP-AAV at the given time point and genotype. **(A)** Wild-type (WT) mice receiving IFNγ-AAV for 4 months (4 m). **(B)** WT mice receiving IFNγ-AAV for 6 months (6 m). **(C)** APP/PS1 receiving IFNγ-AAV for 4 months. **(D)** APP/PS1 receiving IFNγ-AAV for 6 months. *P < 0.05, **P < 0.01, IFNγ-AAV compared to GFP-AAV of that time point and genotype. #*P* < 0.05, ##*P* < 0.01, 4-month IFNγ-AAV compared to 6-month IFNγ-AAV of that genotype. AAV, adeno-associated virus; AARG1, arginase 1; FCGR, Fc gamma receptor; GFP, green fluorescent protein; IFN, interferon; IL, interleukin; SPHK1, sphingosine kinase 1; TGFB, transforming growth factor beta.

**Figure 3:**
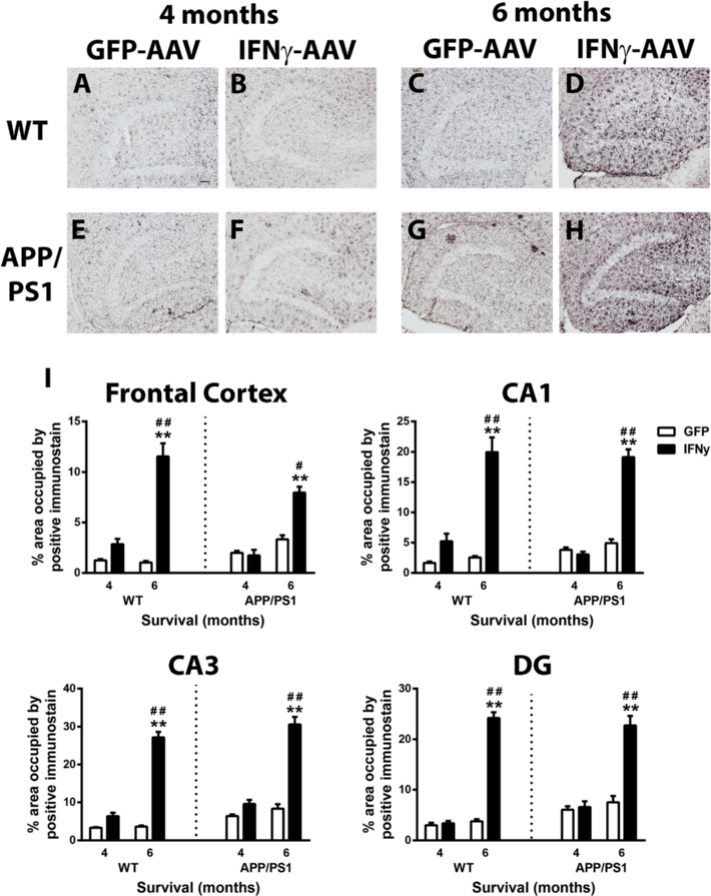
**IFNγ causes an increase in CD11b staining. (A-D)** Dentate gyrus of wild-type (WT) mice receiving GFP-AAV or IFNγ-AAV. **(E-H)** Dentate gyrus of APP/PS1 mice receiving GFP-AAV or IFNγ-AAV. **(I)** Quantification of CD11b in the frontal cortex, CA1, CA3, and DG. **P < 0.01, IFNγ-AAV compared to GFP-AAV at that time point and genotype. #*P* < 0.05, ##*P* < 0.01, 4-month IFNγ-AAV compared to 6-month IFNγ-AAV of that genotype. Scale bar in A is for A-H and is equal to 50 μm. AAV, adeno-associated virus; DG, dentate gyrus, GFP, green fluorescent protein; IFN, interferon.

To determine the neuroinflammatory phenotype, right hippocampal RNA was isolated and real time PCR was performed for several genes specific to an M1, M2a, M2b or M2c macrophage phenotype. Data are shown as fold change compared to GFP-AAV at each time point for each genotype. The GFP-AAV mice showed no significant change in neuroinflammation over time (data not shown). Overall, both WT and APP/PS1 mice responded to the IFNγ-AAV with a robust M1 response. IL-12b was increased in IFNγ-AAV treated mice at 4 and 6 months in both genotypes, and IL-1β was increased at all time points, except 6 months in WT (Figure 2A-D). Additionally, IL-6 and IL-12a showed significant increases at the 6-month (but not 4-month) time point in both WT and APP/PS1 mice (Figure 2B,D). At the 6-month time point, but not at the 4-month time point, M2a gene YM1 was significantly increased in WT and APP/PS1 mice (Figure 2A-D). Also of note are increased M2b markers CD86, FcγR1 and FcγR3 at the 4 and 6-month time point (Figure 2B,D). Table 2 shows the fold-change values for GFP-AAV- and IFNγ-AAV-treated mice. Interestingly, while it may appear that the APP/PS1 mice are less responsive to the IFNγ-AAV, it is actually due to the fact that APP/PS1 mice normally have an inflammatory response to the amyloid deposits in the brain and so the fold-change achieved by the IFNγ-AAV is less than in WT.Next, assessment of CD11b immunohistochemistry in the frontal cortex (images not shown) and the hippocampus (dentate gyrus shown, Figure 3A-H) was performed to determine the effect of IFNγ on microglia. In all four brain regions measured, for both WT and APP/PS1 mice receiving IFNγ-AAV, there were statistically significant increases in CD11b staining, as measured by percent area occupied by positive immunostain, at 6 months compared to 4 months that were not observed in mice receiving GFP-AAV (that is, the treatment-by-time interaction is significant). In the frontal cortex, there was a statistically significant increase in microglial staining at 6 months but not 4 months in both WT and APP/PS1 mice injected with IFNγ-AAV compared to mice receiving GFP-AAV (Figure 3I). At 6 months in WT mice, there were significant increases in staining in the CA1, CA3 and (DG) regions (Figure 3A-D,I). APP/PS1 mice receiving IFNγ-AAV only showed modest, non-significant increases at 4 months (Figure 3E,F,I) but significant increases were observed in all hippocampal regions by 6 months (Figure 3G,H,I).

**Table 2:** Fold change values for real time PCR

	**GFP-AAV**				**IFNγ-AAV**			
	**WT**		**APP/PS1**		**WT**		**APP/PS1**	
**Gene**	**4 mo**	**6 mo**	**4 mo**	**6 mo**	**4 mo**	**6 mo**	**4 mo**	**6 mo**
IL-6	1.5 ± 0.51	1.4 ± 0.45	2.46 ± 1.36	1.16 ± 0.21	5.27 ± 2.8	**92.42 ± 81.72**	12.23 ± 7.43	**15.03 ± 5.81**
IL-1β	1.7 ± 0.72	1.2 ± 0.27	1.42 ± 0.57	1.15 ± 0.23	**26.55 ± 8.8**	**3673.4 ± 3599.1**	77.75 ± 31.53	**96.07 ± 25.74**
IL-12a	1.62 ± 0.68	1.25 ± 0.38	1.75 ± 0.79	1.4 ± 0.44	3.62 ± 1.9	**29.53 ± 0.43**	5.37 ± 3.92	**89.85 ± 60.30**
IL-12b	1.37 ± 0.94	1.29 ± 0.39	1.14 ± 0.43	1.68 ± 0.76	**108.96 ± 36.47**	**109.55 ± 79.82**	**128.58 ± 103.11**	**239.43 ± 77.25**
ARG1	1.95 ± 0.79	1.19 ± 0.34	1.41 ± 0.45	1.88 ± 0.75	0.55 ± 0.09	1.68 ± 0.30	0.49 ± 0.12	2.02 ± 0.36
YM1	1.87 ± 1.06	1.08 ± 0.19	1.31 ± 0.51	2.35 ± 1.17	2.91 ± 1.12	**184.47 ± 174.22**	6.48 ± 4.07	**68.66 ± 23.88**
CD86	1.21 ± 0.3	1.56 ± 0.66	1.09 ± 0.22	1.3 ± 0.93	**21.2 ± 4.97**	**24.9 ± 4.51**	**66.85 ± 43.23**	**19.93 ± 7.52**
FcγR1	1.2 ± 0.28	1.10 ± 0.24	1.12 ± 0.26	1.36 ± 0.55	**13.45 ± 3.05**	**59 ± 34.83**	**9.11 ± 2.61**	**18.44 ± 2.65**
FcγR3	1.49 ± 0.46	1.05 ± 0.15	1.14 ± 0.31	1.27 ± 0.44	**4.9 ± 0.83**	**36.28 ± 10.41**	**4.89 ± 1.61**	**13.64 ± 1.17**
TGFβ1	1.45 ± 0.61	1.29 ± 0.40	1.07 ± 0.19	1.23 ± 0.26	1.58 ± 0.33	**3.53 ± 0.83**	1.95 ± 0.28	1.95 ± 0.74
SPHK1	2.01 ± 1.28	1.07 ± 0.18	1.46 ± 0.60	1.58 ± 0.77	**0.06 ± 0.02**	**8.23 ± 2.69**	**0.19 ± 0.03**	2.21 ± 0.28

Bold font indicates *P* < 0.05 compared to GFP-AAV of that time point and genotype. AAV, adeno-associated virus; ARG1, arginase 1; FcγR, Fc gamma receptor; GFP, green fluorescent protein; IFN, interferon; IL, interleukin; mo, months; SPHK1, sphingosine kinase 1; TGF, transforming growth factor; WT, wild-type.

**Figure 4:**
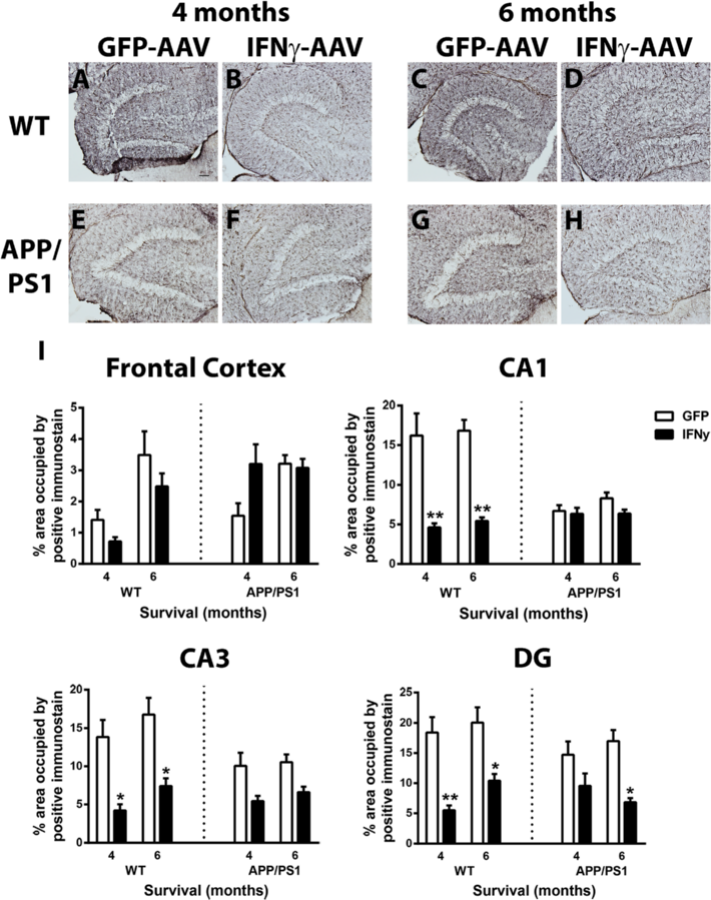
**IFNγ causes a decrease in GFAP staining. (A-D)** Dentate gyrus of wild-type (WT) mice receiving GFP-AAV or IFNγ-AAV. **(E-H)** Hippocampus of APP/PS1 mice receiving GFP-AAV or IFNγ-AAV. **(I)** Quantification of GFAP in the frontal cortex, CA1, CA3, and DG. *P < 0.05, **P < 0.01, IFNγ-AAV compared to GFP-AAV at that time point and genotype. Scale bar in A is for A-H and is equal to 50 μm. AAV, adeno-associated virus; DG, dentate gyrus; GFAP, glial fibrillary acidic protein; GFP, green fluorescent protein; IFN, interferon.

**Figure 5:**
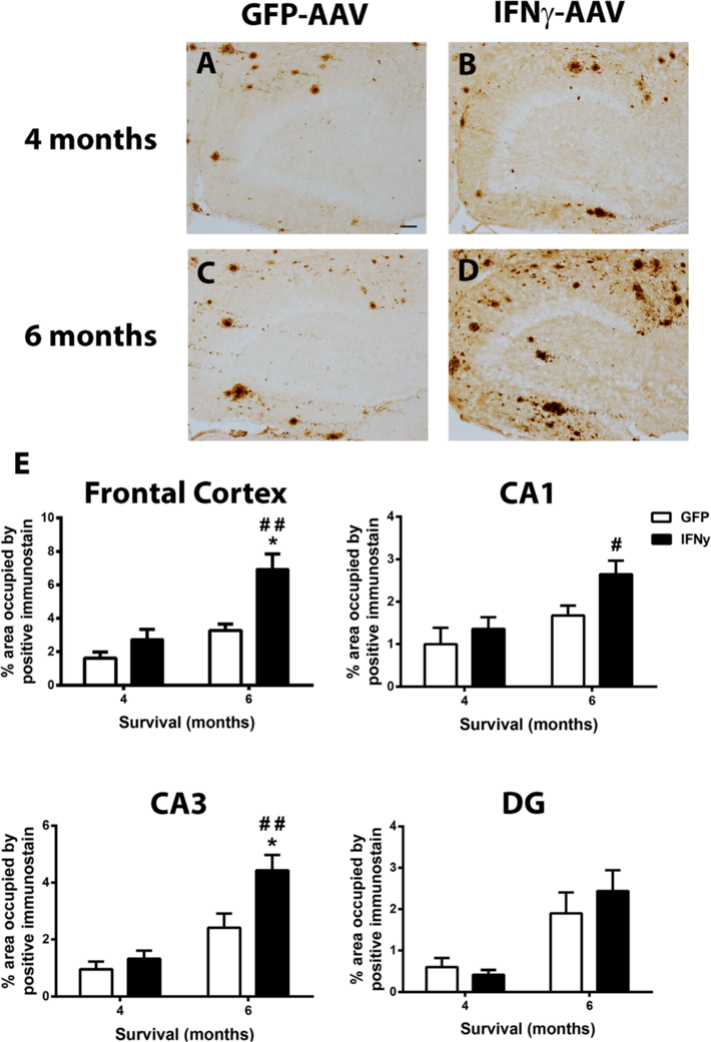
**IFNγ increases Aβ deposition. (A-D)** Dentate gyrus of APP/PS1 mice receiving GFP-AAV or IFNγ-AAV. **(E)** Quantification of Aβ in the frontal cortex, CA1, CA3, and DG. *P < 0.05, IFNγ-AAV compared to GFP-AAV at that time point and genotype. #*P* < 0.05, ##*P* < 0.01, 4-month IFNγ-AAV compared to 6-month IFNγ-AAV of that genotype. Scale bar in A is for A-D and is equal to 50 μm. Aβ, amyloid-beta; AAV, adeno-associated virus; DG, dentate gyrus; GFP, green fluorescent protein; IFN, interferon.

Evaluation of astrogliosis using GFAP immunohistochemistry was also performed on the frontal cortex (images not shown) and the hippocampus (dentate gyrus shown, Figure 4A-H). Distribution of GFAP staining in WT (Figure 4A,C) and APP/PS1 mice (Figure 4E,G) receiving GFP-AAV was typical for age and genotype [24]. Curiously, IFNγ-AAV injection in both the WT and APP/PS1 mice resulted in decreased GFAP-positive staining. We found significantly decreased GFAP staining 4 months after IFNγ-AAV injection in the CA1, CA3, and DG regions of WT mice (Figure 4A,B,I) There were no significant changes in GFAP staining in the CA1, CA3, or DG regions in the APP/PS1 mice at 4 months (Figure 4E,F,I). At 6 months, there was a significant decrease in astrocyte staining in the CA1, CA3 and DG regions in WT (Figure 4C,D,I) and in the DG in APP/PS1 mice (Figure 4G,H,I).Finally, we examined the effect of IFNγ-AAV on Aβ levels in the APP/PS1 mice. Aβ levels were determined by immunohistochemistry and ELISA analysis. Aβ deposition in the GFP-AAV mice showed a typical distribution of APP/PS1 mice at this age both 4 and 6 months post-injection (Figure 5A,C). Immunohistochemical analysis of the frontal cortex (images not shown) and the hippocampus (DG shown, Figure 5A-D) shows no significant change in Aβ deposition 4 months post-injection (Figure 5A,B,E) but, at 6 months post-injection, IFNγ-AAV resulted in increased Aβ deposition in the frontal cortex, CA1 and CA3 regions versus GFP-AAV treated mice. Aβ deposition was elevated significantly at 6 months compared to levels at 4 months in mice receiving IFNγ-AAV in all areas except the DG, where the effect was marginally significant (Figure 5B,D,E).

Biochemical detection of Aβ was performed on PBS soluble and insoluble (formic acid soluble) protein extracts from the left anterior cerebral cortex. Aβ1-38, 1-40 and 1-42 were analyzed by multiplexed ELISA analysis (Table 3). IFNγ-AAV injection significantly increased soluble Aβ1-40 and marginally increased Aβ1-42 levels at 4 months relative to GFP-AAV injection in APP/PS1 mice (Table 3). Soluble Aβ1-38, Aβ1-40 and Aβ1-42 levels significantly increased from 4 to 6 months in both treatment groups at the same rate (that is, the treatment-by-time interactions were not significant), but treatment group differences at 6 months were not significant due to higher variability in the IFNγ-AAV treated mice. Main effects for treatment were significant for soluble Aβ1-38, Aβ1-40 and Aβ1-42. No significant changes due to treatment or time were observed for insoluble Aβ, with the exception of Aβ1-38, where there was a main effect due to time (*P* = 0.024).

**Table 3:** Soluble and insoluble amyloid-beta levels measured by ELISA

		**Soluble (pg/μg protein)**			**Insoluble (pg/μg protein)**		
**AAV injection**	**Survival (months)**	**Aβ1-38**	**Aβ1-40**	**Aβ1-42**	**Aβ1-38**	**Aβ1-40**	**Aβ1-42**
GFP-AAV	4	1.67 ± 0.77	2.97 ± 0.28	12.90 ± 2.24	9.04 ± 1.99	98.70 ± 36.77	903.27 ± 56.1
GFP-AAV	6	3.16 ± 0.75	5.10 ± 0.44	25.37 ± 3.81	23.82 ± 2.83	335.53 ± 64.95	1634.87 ± 397.9
IFNγ-AAV	4	3.68 ± 0.23	**5.41 ± 0.91**	27.17 ± 4.94	20.56 ± 8.48	295.78 ± 87.63	2855.26 ± 1267.9
IFNγ-AAV	6	4.95 ± 0.51	6.57 ± 0.89	38.90 ± 7.35	27.10 ± 4.10	269.72 ± 55.2	2047.32 ± 436.0

Bold indicates *P* < 0.05 compared to GFP-AAV at the same time point. Aβ, amyloid-beta; AAV, adeno-associated virus; ELISA, enzyme-linked immunosorbent assay; IFN, interferon.

## Discussion

Alzheimer’s disease, while characterized by amyloid plaques and neurofibrillary tangles, is associated with a robust neuroinflammatory response. Using the macrophage classification system of M1, M2a, M2b and M2c [6,7] we recently showed that the neuroinflammatory state is highly variable in the early stages of AD [25]. This variability was apparent in a population that was clinically and pathologically indistinguishable suggesting that neuroinflammation may be a source of variability in the Alzheimer’s population. Furthermore, we hypothesize the neuroinflammatory state will influence progression of the disease and also response to therapeutic interventions. To better determine the role these distinct neuroinflammatory phenotypes have on amyloid pathology, we developed AAV vectors to express IFNγ, the initiator of an M1 neuroinflammatory state [26,27], with the goal of chronically polarizing the state of the brain to an M1 state. We then injected this into the brains of APP/PS1 mice and examined their pathological progression 4 and 6 months post-injection. Overall, our data suggest that, while initially we did polarize the phenotype to M1, by 6 months the neuroinflammatory phenotype showed a combination of M1 and M2. Amyloid deposition was unaffected by the M1 phenotype but increased when the combination of inflammatory markers were increased. Whether this increase in Aβ is due to the M2 phenotypes direct actions or indirect actions on the M1 microglia remains unclear.

Macrophage activation can be categorized as M1, M2a, M2b and M2c. M1 is characterized by high IL-12 and low IL-10 while the M2 phenotypes are characterized by high IL-10 and low IL-12 [8]. Additionally, M1 macrophages express high levels of IL-1β, TNFα and IL-6 [6]. M2a macrophages are associated with wound healing and repair gene expression including YM1 and ARG1 [8]. The M2b phenotype is very much like an M1 phenotype with the exception of the IL-10/IL-12 balance and high expression of CD86 [8]. Finally, the M2c state is characterized by high transforming growth factor beta and sphingosine kinase 1 [6,9]. IFNγ, with and without LPS or TNFα, is the typical M1 phenotype stimulus [26,27]. Using BV2 microglial cell cultures, we have previously shown that treatment of BV2 cells with IFNγ results in an M1 phenotype [28]. We hypothesized that a continuous presence of IFNγ would chronically polarize the inflammatory state of the brain to an M1 state. We found that expression of IFNγ through intracranial injection of IFNγ-AAV successfully induced an M1 phenotype at 4 months post-injection, but over time this transitioned to a combined M1 and M2 phenotype by 6 months. Both the early M1 phenotype and the 6 month transition to a mixed phenotype were present in both WT and APP/PS1 mice, indicating that the APP/PS1 mice and WT mice responded similarly to the IFNγ-AAV injection.

CD11b (CR3) has been used as one of a handful of microglial activation markers. In the mouse, CD11b labels ramified resting microglia, while GFAP labels astrocytes and the intensity of the staining is generally increased with inflammatory stimuli [29,30]. There is an assumption in the field that microglial activation and astrogliosis correspond to high levels of pro-inflammatory cytokines such as IL-1β and IL-6. In the current study, we find that CD11b immunostaining is not affected by the IFNγ-AAV injection at the 4-month time point, when the neuroinflammatory state is primarily M1, but is only increased when M2 markers are also increased. We also find that GFAP staining decreases when M2 markers increase even though M1 genes are still high. These data suggest that more careful analysis of neuroinflammatory markers is necessary for appropriate interpretation of data. The CD11b data here would indicate that inflammation is only increased at the 6-month time point, yet we see that at the 4-month time point IL-1β and IL-12 are significantly elevated.

The role of neuroinflammation on amyloid deposition remains controversial. Studies with LPS injection have shown both decreases and increases in amyloid load with neuroinflammation [15,17,31]. Additionally, microglial “activation” has been linked to both increased and decreased amyloid loads [29,31]. Our goal in this study was to determine the influence of an M1 neuroinflammatory phenotype on amyloid deposition. At the 4-month time-point, when M1 neuroinflammation predominates in the brains of mice injected with IFNγ-AAV, amyloid deposition appears unaffected with no significant difference between IFNγ-AAV and GFP-AAV injected mice. We do find increased Aβ deposition at the 6-month time point, but this is in the presence of a mixed neuroinflammatory phenotype. At this time point we see not only M1 markers including IL-12, IL-1β and IL-6, but also the M2a marker YM1. This is not the first time that M1 markers have been associated with unchanging or decreased amyloid deposition [13]. Indeed, anti-Aβ immunotherapy initiates activation of M1 markers [32]. Furthermore, we have previously shown that lithium treatment results in unchanging biochemical levels of Aβ but increased amyloid deposition accompanied by increased expression of M2a markers YM1, ARG1 and IL-1 receptor antagonist [22]. In this study we also see relatively unchanging biochemical levels of Aβ, increased Aβ deposition shown by an increase in Aβ staining and an increase in the expression of M2a markers at 6 months. This increase in Aβ staining at 6 months implies that a mixed neuroinflammatory phenotype increases amyloid burden. While the mechanism is unclear, it appears that an M2a phenotype promotes amyloid deposition in the absence of changing total Aβ levels. Future studies will attempt to elucidate the mechanism of this phenomenon.

Human data from Alzheimer’s patients also shows a similar trend in neuroinflammatory switching. Our laboratory has previously shown that, in early AD, patients are polarized towards either an M1 or an M2a phenotype [25]. By end-stage AD, patients have a combined M1, M2a and M2c phenotype. Also, early AD patients who polarized towards an M2a phenotype had an increased neuritic plaque load compared to those polarized towards an M1 phenotype. This suggests that the transition to a mixed phenotype is associated with disease progression and increased amyloid burden. Future studies on the effects of the neuroinflammatory phenotypes, especially an M2 phenotype, could provide possible therapeutic targets for AD progression and potential biomarkers to determine the stage of AD.

## Conclusions

Expression of IFNγ through AAV successfully induced an M1 phenotype at 4 months that transitioned to a mixed phenotype by 6 months. This transition also appeared with an increase in amyloid burden suggesting that a mixed phenotype, or enhanced expression of M2a and M2c markers, could contribute to increasing amyloid burden and disease progression.

## Abbreviations

Aβ: amyloid-beta; AAV: adeno-associated viral; AD: Alzheimer’s disease; ANOVA: analysis of variance; ARG1: arginase 1; bp: base pair; DG: dentate gyrus; ELISA: enzyme-linked immunosorbent assay; FcγR: Fc gamma receptor; GFAP: glial fibrillary acidic protein; GFP: green fluorescent protein; IFN: interferon; IL: interleukin; LPS: lipopolysaccharide; PBS: phosphate-buffered saline; PCR: polymerase chain reaction; TNF: tumor necrosis factor; WT: wild-type.

## Competing interests

The authors declare they have no competing interests.

## Authors’ contributions

EMW performed the data collection and analysis, interpreted the data and prepared the manuscript. TLS managed the animals in the study and assisted in data collection. ELA analyzed the data. GJP and MDM developed the AAV vectors. HMB assisted in revision of the manuscript. KB and AG assisted in tissue processing and data collection. DMW conceived of the studies, analyzed and interpreted the data, and edited the final manuscript. All authors have read and approved the final version of this manuscript.
